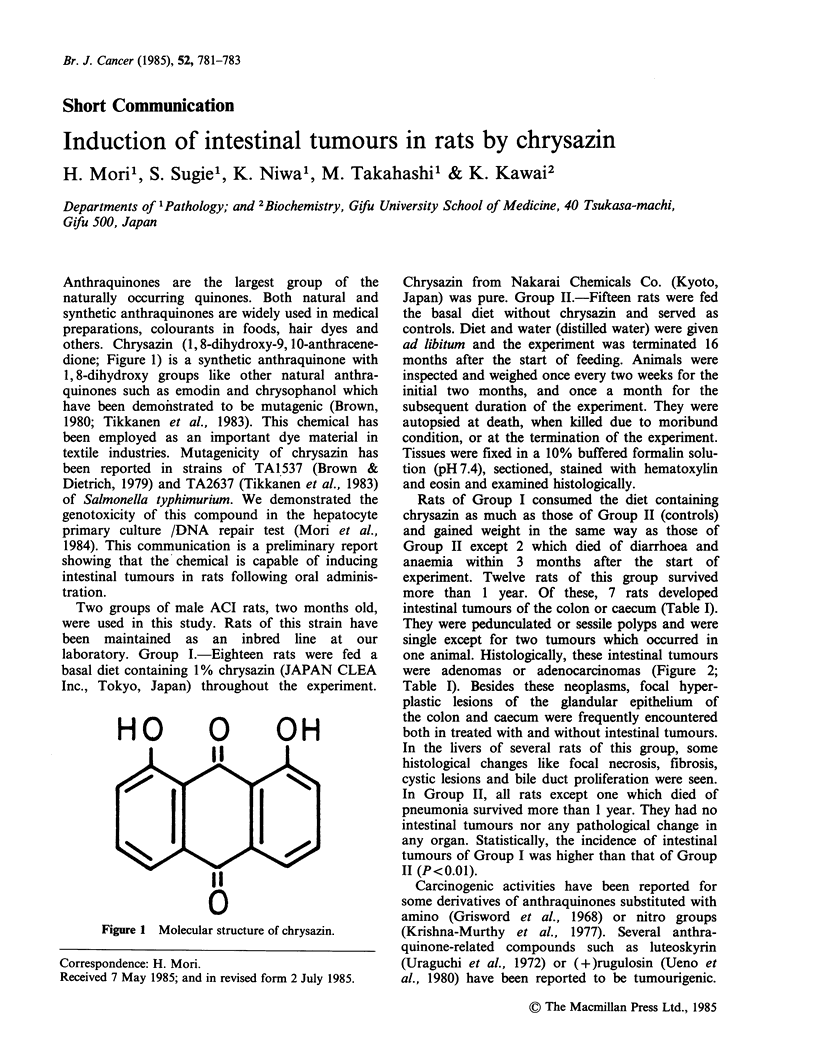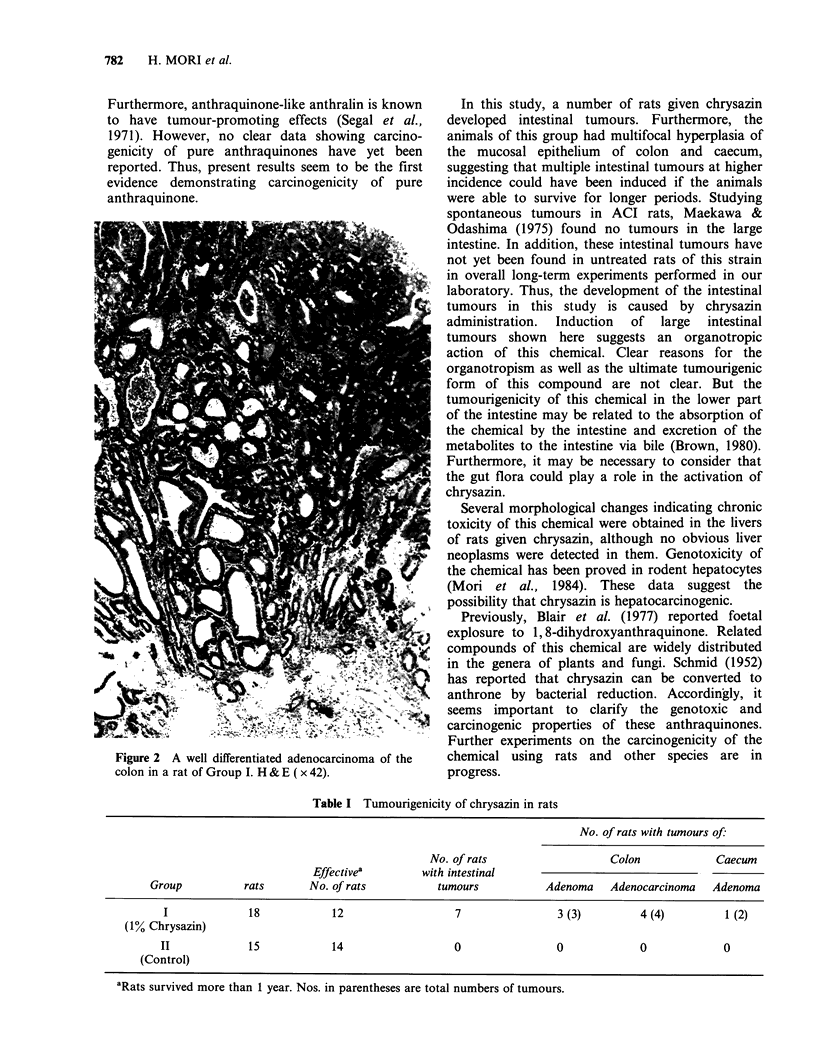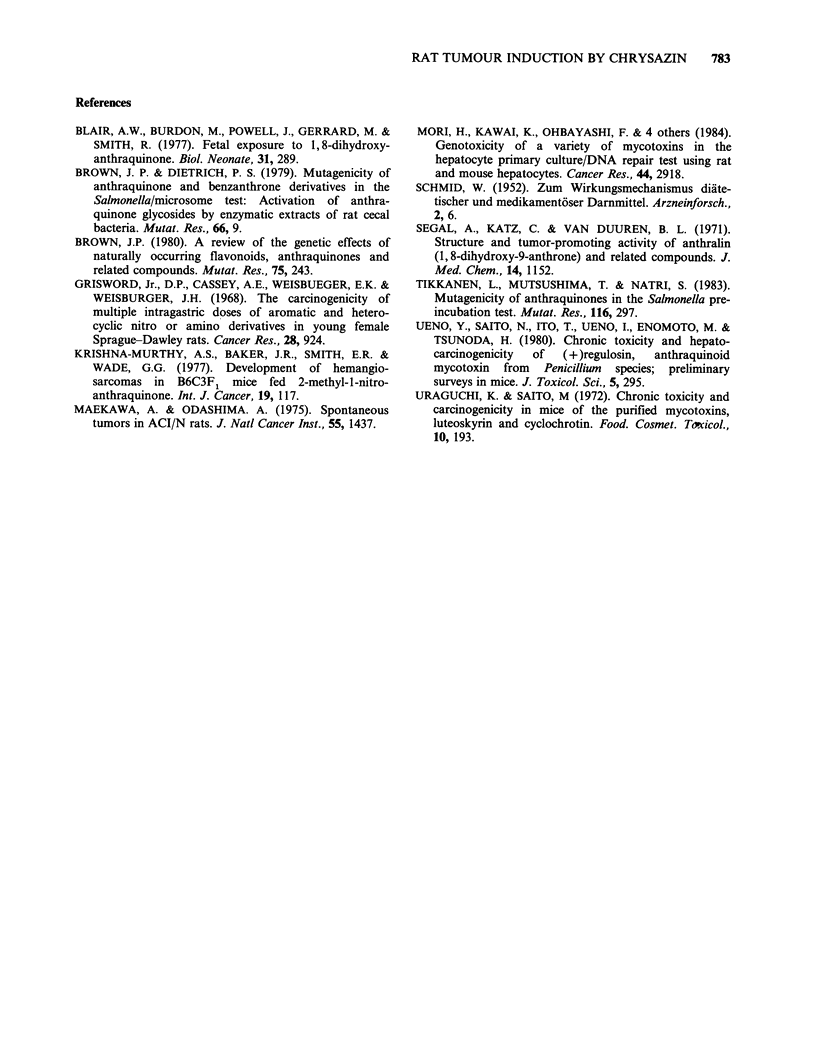# Induction of intestinal tumours in rats by chrysazin.

**DOI:** 10.1038/bjc.1985.257

**Published:** 1985-11

**Authors:** H. Mori, S. Sugie, K. Niwa, M. Takahashi, K. Kawai

## Abstract

**Images:**


*Br. J. Cancer* (1985), **52**, 781–783

### Short Communication

### Induction of intestinal tumours in rats by chrysazin

H. Mori[1], S. Sugie[1], K. Niwa[1], M. Takahashi[1] & K. Kawai[2]

*Departments of [1]Pathology; and [2]Biochemistry, Gifu University School of Medicine, 40 Tsukasa-machi, Gifu 500, Japan*

Anthraquinones are the largest group of the naturally occurring quinones. Both natural and synthetic anthraquinones are widely used in medical preparations, colourants in foods, hair dyes and others. Chrysazin (1,8-dihydroxy-9,10-anthracenedione; Figure 1) is a synthetic anthraquinone with 1,8-dihydroxy groups like other natural anthraquinones such as emodin and chrysophanol which have been demonstrated to be mutagenic (Brown, 1980; Tikkanen *et al.*, 1983). This chemical has been employed as an important dye material in textile industries. Mutagenicity of chrysazin has been reported in strains of TA1537 (Brown & Dietrich, 1979) and TA2637 (Tikkanen *et al.*, 1983) of *Salmonella typhimurium*. We demonstrated the genotoxicity of this compound in the hepatocyte primary culture /DNA repair test (Mori *et al.*, 1984). This communication is a preliminary report showing that the chemical is capable of inducing intestinal tumours in rats following oral administration.

Two groups of male ACI rats, two months old, were used in this study. Rats of this strain have been maintained as an inbred line at our laboratory. Group I.—Eighteen rats were fed a basal diet containing 1% chrysazin (JAPAN CLEA Inc., Tokyo, Japan) throughout the experiment.

**Figure 1** Molecular structure of chrysazin.

Correspondence: H. Mori.
Received 7 May 1985; and in revised form 2 July 1985.

Chrysazin from Nakarai Chemicals Co. (Kyoto, Japan) was pure. Group II.—Fifteen rats were fed the basal diet without chrysazin and served as controls. Diet and water (distilled water) were given *ad libitum* and the experiment was terminated 16 months after the start of feeding. Animals were inspected and weighed once every two weeks for the initial two months, and once a month for the subsequent duration of the experiment. They were autopsied at death, when killed due to moribund condition, or at the termination of the experiment. Tissues were fixed in a 10% buffered formalin solution (pH 7.4), sectioned, stained with hematoxylin and eosin and examined histologically.

Rats of Group I consumed the diet containing chrysazin as much as those of Group II (controls) and gained weight in the same way as those of Group II except 2 which died of diarrhoea and anaemia within 3 months after the start of experiment. Twelve rats of this group survived more than 1 year. Of these, 7 rats developed intestinal tumours of the colon or caecum (Table I). They were pedunculated or sessile polyps and were single except for two tumours which occurred in one animal. Histologically, these intestinal tumours were adenomas or adenocarcinomas (Figure 2; Table I). Besides these neoplasms, focal hyperplastic lesions of the glandular epithelium of the colon and caecum were frequently encountered both in treated with and without intestinal tumours. In the livers of several rats of this group, some histological changes like focal necrosis, fibrosis, cystic lesions and bile duct proliferation were seen. In Group II, all rats except one which died of pneumonia survived more than 1 year. They had no intestinal tumours nor any pathological change in any organ. Statistically, the incidence of intestinal tumours of Group I was higher than that of Group II ($P<0.01$).

Carcinogenic activities have been reported for some derivatives of anthraquinones substituted with amino (Grisword *et al.*, 1968) or nitro groups (Krishna-Murthy *et al.*, 1977). Several anthraquinone-related compounds such as luteoskyrin (Uraguchi *et al.*, 1972) or (+)rugulosin (Ueno *et al.*, 1980) have been reported to be tumourigenic.

© The Macmillan Press Ltd., 1985

Furthermore, anthraquinone-like anthralin is known to have tumour-promoting effects (Segal *et al.*, 1971). However, no clear data showing carcinogenicity of pure anthraquinones have yet been reported. Thus, present results seem to be the first evidence demonstrating carcinogenicity of pure anthraquinone.

**Figure 2** A well differentiated adenocarcinoma of the colon in a rat of Group I. H & E ( × 42).

In this study, a number of rats given chrysazin developed intestinal tumours. Furthermore, the animals of this group had multifocal hyperplasia of the mucosal epithelium of colon and caecum, suggesting that multiple intestinal tumours at higher incidence could have been induced if the animals were able to survive for longer periods. Studying spontaneous tumours in ACI rats, Maekawa & Odashima (1975) found no tumours in the large intestine. In addition, these intestinal tumours have not yet been found in untreated rats of this strain in overall long-term experiments performed in our laboratory. Thus, the development of the intestinal tumours in this study is caused by chrysazin administration. Induction of large intestinal tumours shown here suggests an organotropic action of this chemical. Clear reasons for the organotropism as well as the ultimate tumourigenic form of this compound are not clear. But the tumourigenicity of this chemical in the lower part of the intestine may be related to the absorption of the chemical by the intestine and excretion of the metabolites to the intestine via bile (Brown, 1980). Furthermore, it may be necessary to consider that the gut flora could play a role in the activation of chrysazin.

Several morphological changes indicating chronic toxicity of this chemical were obtained in the livers of rats given chrysazin, although no obvious liver neoplasms were detected in them. Genotoxicity of the chemical has been proved in rodent hepatocytes (Mori *et al.*, 1984). These data suggest the possibility that chrysazin is hepatocarcinogenic.

Previously, Blair *et al.* (1977) reported foetal explosure to 1,8-dihydroxyanthraquinone. Related compounds of this chemical are widely distributed in the genera of plants and fungi. Schmid (1952) has reported that chrysazin can be converted to anthrone by bacterial reduction. Accordingly, it seems important to clarify the genotoxic and carcinogenic properties of these anthraquinones. Further experiments on the carcinogenicity of the chemical using rats and other species are in progress.

**Table I** Tumourigenicity of chrysazin in rats

| Group | rats | *Effective*[a] *No. of rats* | *No. of rats with intestinal tumours* | *No. of rats with tumours of:* Colon Adenoma | Colon Adenocarcinoma | Caecum Adenoma |
|---|---|---|---|---|---|---|
| I (1% Chrysazin) | 18 | 12 | 7 | 3 (3) | 4 (4) | 1 (2) |
| II (Control) | 15 | 14 | 0 | 0 | 0 | 0 |

[a]Rats survived more than 1 year. Nos. in parentheses are total numbers of tumours.

### References

BLAIR, A.W., BURDON, M., POWELL, J., GERRARD, M. & SMITH, R. (1977). Fetal exposure to 1,8-dihydroxyanthraquinone. *Biol. Neonate,* **31,** 289.

BROWN, J. P. & DIETRICH, P. S. (1979). Mutagenicity of anthraquinone and benzanthrone derivatives in the *Salmonella*/microsome test: Activation of anthraquinone glycosides by enzymatic extracts of rat cecal bacteria. *Mutat. Res.,* **66,** 9.

BROWN, J.P. (1980). A review of the genetic effects of naturally occurring flavonoids, anthraquinones and related compounds. *Mutat. Res.,* **75,** 243.

GRISWORD, Jr., D.P., CASSEY, A.E., WEISBUEGER, E.K. & WEISBURGER, J.H. (1968). The carcinogenicity of multiple intragastric doses of aromatic and heterocyclic nitro or amino derivatives in young female Sprague–Dawley rats. *Cancer Res.,* **28,** 924.

KRISHNA-MURTHY, A.S., BAKER, J.R., SMITH, E.R. & WADE, G.G. (1977). Development of hemangiosarcomas in $B6C3F_1$ mice fed 2-methyl-1-nitroanthraquinone. *Int. J. Cancer,* **19,** 117.

MAEKAWA, A. & ODASHIMA. A. (1975). Spontaneous tumors in ACI/N rats. *J. Natl Cancer Inst.,* **55,** 1437.

MORI, H., KAWAI, K., OHBAYASHI, F. & 4 others (1984). Genotoxicity of a variety of mycotoxins in the hepatocyte primary culture/DNA repair test using rat and mouse hepatocytes. *Cancer Res.,* **44,** 2918.

SCHMID, W. (1952). Zum Wirkungsmechanismus diätetischer und medikamentöser Darnmittel. *Arzneinforsch.,* **2,** 6.

SEGAL, A., KATZ, C. & VAN DUUREN, B. L. (1971). Structure and tumor-promoting activity of anthralin (1,8-dihydroxy-9-anthrone) and related compounds. *J. Med. Chem.,* **14,** 1152.

TIKKANEN, L., MUTSUSHIMA, T. & NATRI, S. (1983). Mutagenicity of anthraquinones in the *Salmonella* preincubation test. *Mutat. Res.,* **116,** 297.

UENO, Y., SAITO, N., ITO, T., UENO, I., ENOMOTO, M. & TSUNODA, H. (1980). Chronic toxicity and hepatocarcinogenicity of (+)regulosin, anthraquinoid mycotoxin from *Penicillium* species; preliminary surveys in mice. *J. Toxicol. Sci.,* **5,** 295.

URAGUCHI, K. & SAITO, M (1972). Chronic toxicity and carcinogenicity in mice of the purified mycotoxins, luteoskyrin and cyclochrotin. *Food. Cosmet. Toxicol.,* **10,** 193.